# Rudimentary Horn Pregnancy Diagnosed after Laparotomy

**DOI:** 10.1155/2020/5816487

**Published:** 2020-07-23

**Authors:** Kurabachew Mengistu, Tufa Bobe, Gashaw Tilahun, Kibru Kifle, Dereje Geleta

**Affiliations:** ^1^Department of Anesthesiology, College of Medicine and Health Sciences, Hawassa University, Hawassa, Ethiopia; ^2^Department of Obstetrics and Gynaecology, College of Medicine and Health Sciences, Madda Wallabu University, Robe, Ethiopia; ^3^Department of Obstetrics and Gynaecology, College of Medicine and Health Sciences, Hawassa University, Hawassa, Ethiopia; ^4^School of Public and Environmental Health, College of Medicine and Health Sciences, Hawassa University, Hawassa, Ethiopia

## Abstract

Müllerian abnormalities are present in 0.17% of fertile women and 3.5% of infertile women, and a unicornuate uterus is observed in 0.4% of women. The uterus is normally formed during embryogenesis by the fusion of the two Müllerian ducts. If one of the ducts does not develop, only one Müllerian duct contributes to the uterine development. We report a case of Gravida II, abortion I referred from a primary hospital with a referral paper and sonography stating she had IUFD. She had regular antenatal care follow-up at the primary hospital and had 8 months of amenorrhea. Our ultrasound assessment confirmed the intrauterine fetal demise, but the rudimentary horn pregnancy was missed. Repeated attempts at the induction of labor were tried but unsuccessful. The diagnosis was confirmed at laparotomy. She underwent cesarean section with right intact rudimentary horn removal. A nonviable male fetus with birth weight of 1.2 kg was delivered. Women with this abnormality are asymptomatic and unaware of having a unicornuate uterus. Abdominal pain is the most common presenting symptom with the rudimentary horn, but communicating horn pregnancy is generally asymptomatic in early pregnancy. Early awareness of this rare clinical condition is so crucial especially in developing countries where the availability of new technologies is scarce to explore uterine abnormalities. The patient had uneventful postoperative recovery and was discharged after 3 postoperative days.

## 1. Introduction

Congenital malformations of the female genital tract are defined as deviations from normal anatomy resulting from embryological maldevelopment of the Müllerian or paramesonephric ducts. They represent a rather common benign condition with a prevalence of 4–7% [1].

The incidence of pregnancy occurring in a rudimentary horn of the unicornuate uterus is very rare ranging from 1 in 75,000 to 150,000 pregnancies [2].

As most of the rudimentary horns are asymptomatic, only 8% of rudimentary horn pregnancies are diagnosed before the symptoms appear [3].

Expert ultrasonography can reveal some Müllerian anomalies, but confirmation requires magnetic resonance imaging [4].

Cases with rudimentary horn pregnancy usually result in the rupture of the horn in the second or third trimester, typically between the 10^th^ and 20^th^ week of gestation, although a rupture has been reported at 34 weeks [5].

This is a case demonstrating a communicating rudimentary horn connected to a unicornuate uterus, where the diagnosis was revealed after surgery.

## 2. Case Presentation

A 22-year-old Gravida II, abortion I came to our institution on referral from a primary hospital with a referral paper and sonography showing that she had IUFD. She had a spontaneous abortion for which dilation and curettage (D&C) was performed at the primary hospital 16 months back. She had no remarkable medical and surgical histories. She had regular antenatal care follow-up at the primary hospital and had 8 months of amenorrhea.

Upon admission, the patient was clinically asymptomatic and stable, showing no uterine contractions and no cervical dilatation. On abdominal examination, the uterus was enlarged equivalent in size to 30 weeks of pregnancy.

On examination, her BP was 120/75 mm of Hg, pulse 85/minute, temperature 36.5°C, and respiratory rate 22/minute. FHS was absent

Obstetric ultrasonography revealed a singleton, intrauterine fetal demise with a gestational age of 32 weeks, estimated fetal weight of 1.3 kg, and negative fetal heartbeat. The placenta was fundal and anteriorly. The cervix was closed, uneffaced, and posterior. All laboratory investigations were normal.

Initially, she was administered 50 mcg of vaginal misoprostol at 6-hour intervals, without response after the 6^th^ dose. Then, she was given an infusion of normal saline 0.9% with 5 IU of oxytocin, followed by increasing concentrations of the same infusion. Just then, there was no cervical dilation, despite the onset of contractions.

Cervical effacement with no dilatation was seen. Finally, a Foley catheter was used. All attempts were unsuccessful. The patient was counselled and underwent a cesarean section horizontally under spinal anaesthesia.

**Figure 1:**
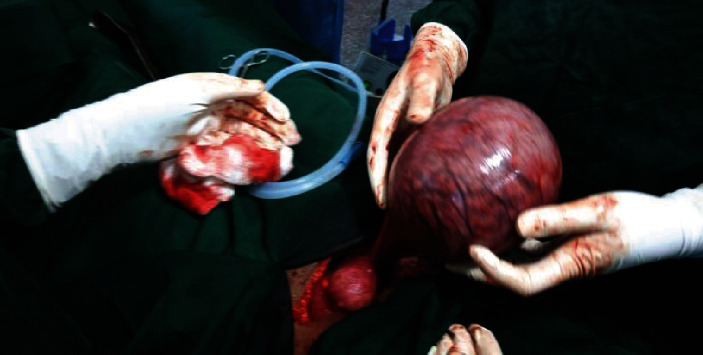
Intraoperative finding of intact rudimentary horn pregnancy.

**Figure 2:**
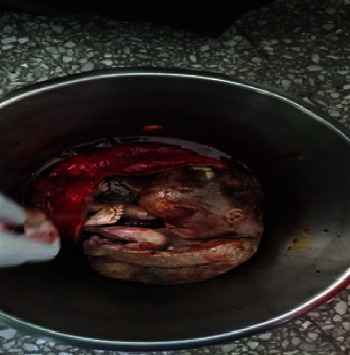
Excised rudimentary horn with the dead fetus.

The intraoperative finding was intact rudimentary horn pregnancy. Clamps were applied over the fibrous band on the medial side and the fallopian tube and ovarian and round ligaments on the lateral side; the accessory horn was excised with the dead fetus; and right salpingo-oophorectomy was performed (Figures 1 and 2).

On releasing the forceps applied to the fibrous band and probing, a canal was detected between the rudimentary horn and the uterine cavity at the level of the isthmus. The round ligament, ovarian ligament, and tube were connected to the right cornua of the uterus.

The patient was advised to follow up. The patient had uneventful postoperative recovery and was discharged after 3 postoperative days.

## 3. Discussion

A unicornuate uterus with a rudimentary horn is a congenital uterine anomaly which results from the developmental arrest of the two Müllerian ducts with the simultaneous incomplete fusion of the opposite side [6].

The unicornuate uterus with rudimentary horn may have a cavity that is either in communication with (type A1a) or sealed off from (type A1b) the primary uterine cavity, or it may have failed to canalize entirely and is without a cavity (type A2, [7]).

When a rudimentary horn is present, it is the noncavitary type in 33% of cases, the cavitary but noncommunicating type in 22% of cases, and the cavitary and communicating type in 10% of cases [8].

Most cases of rudimentary horn pregnancy (RHP) provide a diagnostic challenge and are diagnosed after rupture, which leads to emergency surgery, blood transfusions, and increased morbidity [8].

Diagnosis is challenging in this malformation due to a limited field of sonographic view compared to other diagnostic imaging modalities and lateral deviation of the rudimentary horn [9].

Sonographic sensitivity is only 26%, and as the pregnancy advances, the specificity goes down [10].

The common misdiagnosis on ultrasound includes a bicornuate uterus with pregnancy in one horn, uterus didelphys, abdominal pregnancy, or even normal intrauterine pregnancy with an adnexal mass undergoing torsion [11].

It is shown that 62% of the rudimentary horn is preferentially placed on the right, which was revealed in our case report because the left Müller's canal progresses more caudally than the right [9].

Our patient had no renal abnormalities despite approximately 38% of patients having coexisting renal abnormalities. Unilateral renal agenesis is most commonly found; this is always ipsilateral with the rudimentary horn [12].

The literature presently supports prompt excision of the horn, pregnancy, and ipsilateral once a rudimentary horn pregnancy has been diagnosed [13].

## 4. Conclusion

Early awareness of this rare clinical condition is so crucial especially in developing countries where the availability of new technologies is scarce to explore uterine abnormalities. A high index of suspicion should be made if there is no response to repeated failed attempts at the induction of labor.
